# Bufalin Induces Mitochondria-Dependent Apoptosis in Pancreatic and Oral Cancer Cells by Downregulating hTERT Expression via Activation of the JNK/p38 Pathway

**DOI:** 10.1155/2015/546210

**Published:** 2015-12-10

**Authors:** Xin Tian, Shundong Dai, Jing Sun, Shenyi Jiang, Chengguang Sui, Fandong Meng, Yan Li, Liye Fu, Tao Jiang, Yang Wang, Jia Su, Youhong Jiang

**Affiliations:** ^1^Molecular Oncology Laboratory of Cancer Research Institute, The First Affiliated Hospital of China Medical University, Shenyang 110001, China; ^2^Department of Pathology, The First Affiliated Hospital and College of Basic Medical Sciences of China Medical University, Shenyang 110001, China; ^3^Institute of Pathology and Pathophysiology, Shenyang 110001, China; ^4^Department of Immunology and Biotherapy, Liaoning Cancer Hospital and Institute, Shenyang 110042, China; ^5^Department of Rheumatology, The First Affiliated Hospital of China Medical University, Shenyang 110001, China

## Abstract

Bufalin, a digoxin-like active component of the traditional Chinese medicine Chan Su, exhibits potent antitumor activities in many human cancers. Bufalin induces mitochondria-dependent apoptosis in cancer cells, but the detailed molecular mechanisms are largely unknown. hTERT, the catalytic subunit of telomerase, protects against mitochondrial damage by binding to mitochondrial DNA and reducing mitochondrial ROS production. In the present study, we investigated the effects of bufalin on the cell viability, ROS production, DNA damage, and apoptosis of CAPAN-2 human pancreatic and CAL-27 human oral cancer cells. Bufalin reduced CAPAN-2 and CAL-27 cell viability with IC_50_ values of 159.2 nM and 122.6 nM, respectively. The reduced cell viability was accompanied by increased ROS production, DNA damage, and apoptosis and decreased expression of hTERT. hTERT silencing in CAPAN-2 and CAL-27 cells by siRNA resulted in increased caspase-9/-3 cleavage and DNA damage and decreased cell viability. Collectively, these data suggest that bufalin downregulates hTERT to induce mitochondria-dependent apoptosis in CAPAN-2 and CAL-27 cells. Moreover, bufalin increased the phosphorylation of JNK and p38-MAPK in CAPAN-2 and CAL-27 cells, and blocking the JNK/p38-MAPK pathway using the JNK inhibitor SP600125 or the p38-MAPK inhibitor SB203580 reversed bufalin-induced hTERT downregulation. Thus, the JNK/p38 pathway is involved in bufalin-induced hTERT downregulation and subsequent induction of apoptosis by the mitochondrial pathway.

## 1. Introduction

Bufalin is a digoxin-like active component of Chan Su, a traditional Chinese medicine produced from the skin and parotid venom glands of the toad [1]. Bufalin exhibits potent antitumor activities in many human cancers such as leukemia [2, 3], hepatocellular carcinoma [4, 5], gastric cancer [6], and colorectal cancer [7]. Moreover, bufalin sensitizes drug-resistant cancers to various chemotherapeutic agents [8, 9]. These anticancer activities of bufalin are mainly attributed to induction of cancer cell apoptosis [10] and inhibition of cancer cell migration and invasion [11, 12]. Mechanism studies in a number of cancer cell lines have shown that bufalin induces apoptosis through activation of the mitochondria-dependent pathway [13–15]; however, detailed molecular mechanisms involved are largely unclear.

Human telomerase reverse transcriptase (hTERT) is the catalytic subunit of the enzyme telomerase, a ribonucleoprotein polymerase that maintains the length of the telomere. Telomerase lengthens telomeres in DNA strands, thereby allowing senescent cells that would otherwise become postmitotic and undergo apoptosis to exceed the Hayflick limit and become potentially immortal, as is often the case with cancerous cells [16]. Increased telomerase activity (TA) is found in >90% of human cancer cells through genetic and epigenetic alterations [17, 18], and the transcriptional regulation of the hTERT gene is a major mechanism involved [19]. Moreover, recent research progress has indicated that hTERT exerts several telomere-independent effects on cell transformation, proliferation, mitochondrial function, cell survival, the DNA damage response, and the regulation of gene expression [20]. Specifically, hTERT overexpression alleviates intracellular ROS production, improves mitochondrial function, and inhibits ROS-mediated apoptosis in cancer cells [21, 22]. hTERT contains a mitochondrial localization signal peptide that targets hTERT to the mitochondria. Mitochondrial hTERT protects against mitochondrial damage by binding to mitochondrial DNA, increasing respiratory chain activity, and decreasing mitochondrial ROS production [23]. Therefore, targeting hTERT has been proposed as a novel strategy for cancer therapy [24].

Although Bufalin has been studied in many cancer cells, however, its role in pancreatic and oral cancer remains largely unknown. In the present study, we investigated the effects of bufalin on the viability and apoptosis, as well as DNA damage and ROS production of the human pancreas cancer cell line CAPAN-2 and the human oral cancer cell line CAL-27. We also investigated the role of hTERT in bufalin-induced effects and the molecular mechanisms involved. Our results indicate that bufalin induces ROS production and mitochondria-dependent apoptosis in CAPAN-2 and CAL-27 cells. These effects were mediated by the downregulation of hTERT expression via the JNK/p38 pathway.

## 2. Materials and Methods

### 2.1. Cell Culture and Treatment

The CAPAN-2 human pancreatic cancer cell line (HTB-80) and the CAL-27 human oral cancer cell line (CRL-2095) were purchased from the American Type Culture Collection (USA). The CAPAN-2 cells were cultured in RPMI-1640 medium supplemented with 10% FBS. The CAL-27 cells were grown in DMEM supplemented with 10% FBS, 100 U/mL penicillin, and 100 *μ*g/mL streptomycin. Both cell lines were maintained at 37°C and 5% CO_2_ in a humidified incubator. Cells were treated with bufalin (Sigma-Aldrich Corp., USA) at the indicated concentrations as in each experiment. Buffer solution containing 0.1% DMSO was used as vehicle control.

### 2.2. Cell Viability Assay

The cell viability was determined using the 3-(4,5-dimethylthiazol-2-yl)-2,5-diphenyl-tetrazolium bromide (MTT, Sigma-Aldrich Corp., St. Louis, MO, USA) assay. Briefly, 5 × 10^3^ cells per well were seeded in 96-well culture plates. After overnight incubation, the cells were treated with bufalin at the indicated concentrations for the indicated times as in each experiment. The cells were subsequently incubated with a final concentration of 0.5 mg/mL MTT for 4 h and the resulting formazan crystals were dissolved in 0.2 *μ*L of dimethyl sulfoxide (DMSO). The quantity of formazan was measured by recording changes in absorbance at 570 nm on a spectrophotometer (Bio-Rad Labs, Sunnyvale, CA). Results were expressed as percentage of the control. The half maximal inhibitory concentration (IC_50_) values of bufalin were calculated by nonlinear regression.

### 2.3. Annexin V Fluorescein Staining Assay

The apoptotic cells were detected using an annexin V–FITC staining kit (BD Pharmingen, CA, USA) following the manufacturer's instructions. Briefly, the cells were harvested, washed with phosphate-buffered saline (PBS), and incubated in 500 *μ*L of binding buffer (PH 7.5, 10 mM HEPES, 2.5 mM CaCl_2_, and 140 mM NaCl) containing annexin V–FITC for 30 min in the dark. After 400 *μ*L of binding buffer was added to stop the reaction, cells were subjected to analysis on a flow cytometer (FACScan, Becton Dickinson, USA).

### 2.4. Determination of Intracellular ROS

Intracellular ROS levels were determined by the oxidation of 2-,7-dichloro-fluorescein diacetate (DCFH-DA) to DCF as previously described [25]. Briefly, the cells were harvested, washed with PBS, and incubated with 50 *μ*mol/L DCFH-DA for 10 minutes at 37°C in the dark. The cells were then washed again with PBS, resuspended in DMEM, and subjected to analysis on a flow cytometer (FACScan, Becton Dickinson, USA).

### 2.5. Comet Assay for DNA Damage

The DNA damage of cells was assessed using the comet assay as previously described [26] with minor modifications. The DNA migration gels were read on a fluorescence microscope (Leica Microsystems Inc., USA). The complete comet, including the head and tail portions, was interpreted using the NIS-Elements F 2.20 image analysis software. The extent of DNA damage was assessed by the comet tail length measured from the trailing edge of the nucleus to the leading edge of the tail. The results are presented as mean ± SD of the median tail moment.

### 2.6. Telomerase Activity Assay

Telomerase activity was detected using the TRAP Telo TAGGG PCR enzyme-linked immunosorbent assay kit from Roche (Mannheim, Germany) following the manufacturer's instructions. 1 *μ*g of 50 bp DNA ladder (Invitrogen Life Technologies, Gaithersburg, MD) was used for molecular weight determination.

### 2.7. Design and Transfection of Small Interfering RNA (siRNA)

Two short hairpin RNAs (shRNAs) specifically targeting the hTERT (shRNA1, GCATTGGAATCAGACAGCACT (sense), shRNA2, TGAGGCCTGAGTGAGTGTTTG (sense)), and a scramble shRNA (GACCTGTACGCCAACACAGTG (sense)) were synthesized by Genepharma (Shanghai, P. R. China). The hTERT shRNA and scramble shRNA sequences were each cloned into the pGCsilencer expressing plasmid (Genechem, Shanghai, P. R. China) and transiently transfected into CAPAN-2 and CAL-27 cells for 48 h using Lipofectamine 2000 reagent (Invitrogen, USA) following the manufacturer's instructions.

### 2.8. Real-Time PCR (RT-PCR)

Total RNA was extracted using the Qiagen RNeasy Mini Kit (Qiagen, Inc., Valencia, CA, USA). cDNA was synthesized using High Capacity cDNA Reverse Transcription Kit (Invitrogen Life Technologies) following the manufacturer's instructions. Real-time PCR (RT-PCR) was performed using SYBR-Green PCR Master Mix (Takara, Tokyo, Japan) on 7300 real-time PCR system (Applied Biosystems, Carlsbad, CA, USA). Primers specific for hTERT used in the RT-PCR were as follows: forward, 5′-CAT GGA CTA CGT CGT GGG AG-3′; reverse, 5′-CCT GTG GAT ATC GTC CAG GC-3′. Primers for *β*-actin were as follows: forward, 5′-CTT CCA GCC TTC CTT CCT GG-3′; reverse, 5′-TTC TGC ATC CTG TCG GCA AT-3′. Data were normalized to *β*-actin levels (internal control).

### 2.9. Western Blotting

The lysed cell homogenates were centrifuged at 13,000 ×g for 10 min at 4°C to remove cell debris. Total proteins were collected and the protein concentrations were determined using the Bradford method. Proteins (20 *μ*g) were separated on 10% SDS-PAGE and transferred to polyvinyl difluoride membranes (Millipore, Bedford, MA). Membranes were blocked with 5% milk in TBS-T (TBS containing Tween 20) for 30 min and incubated with primary antibodies overnight at 4°C. Primary antibodies were all obtained from Abcam (Cambridge, MA) and used at the following dilutions: hTERT (1 : 1000), phospho-JNK1 and phospho-JNK2 (pT183 and pY185, resp.; 1 : 1000), NK1/2 (1 : 500), phospho-c-Jun (pS63; 1 : 5000), c-Jun (1 : 1000), phospho-p38 (pT180; 1 : 1000), p38 (1 : 200), pro-caspase-9 (1 : 2000), cleaved caspase-9 (1 : 2000), pro-caspase-3 (1 : 1000), cleaved caspase-3 (1 : 500), GAPDH (1 : 10000), and *β*-actin (1 : 2500). After washing three times with TBS-T for 10 min, membranes were incubated with goat polyclonal anti-rabbit IgG-H&L-preadsorbed (HRP) secondary antibody at room temperature for 1 h. Protein bands were visualized by chemiluminescent detection and the densitometric values were determined using a gel image analysis system (Bio-Rad, Hercules, CA). Data were normalized to GAPDH or *β*-actin.

### 2.10. Statistical Analysis

All results are presented as mean ± SD from at least three independent experiments. Data were analyzed using Graphpad Prism 5 software (GraphPad Software Inc., LaJolla, USA). Results of different treatment groups were compared using two-tailed Student's *t*-test or* post hoc* Bonferroni's test. Differences with a *p* value less than 0.05 were considered statistically significant.

## 3. Results

### 3.1. Bufalin Increases Intracellular ROS Levels and Induces Cell Death and Apoptosis in CAPAN-2 and CAL-27 Cells

**Figure 1:**
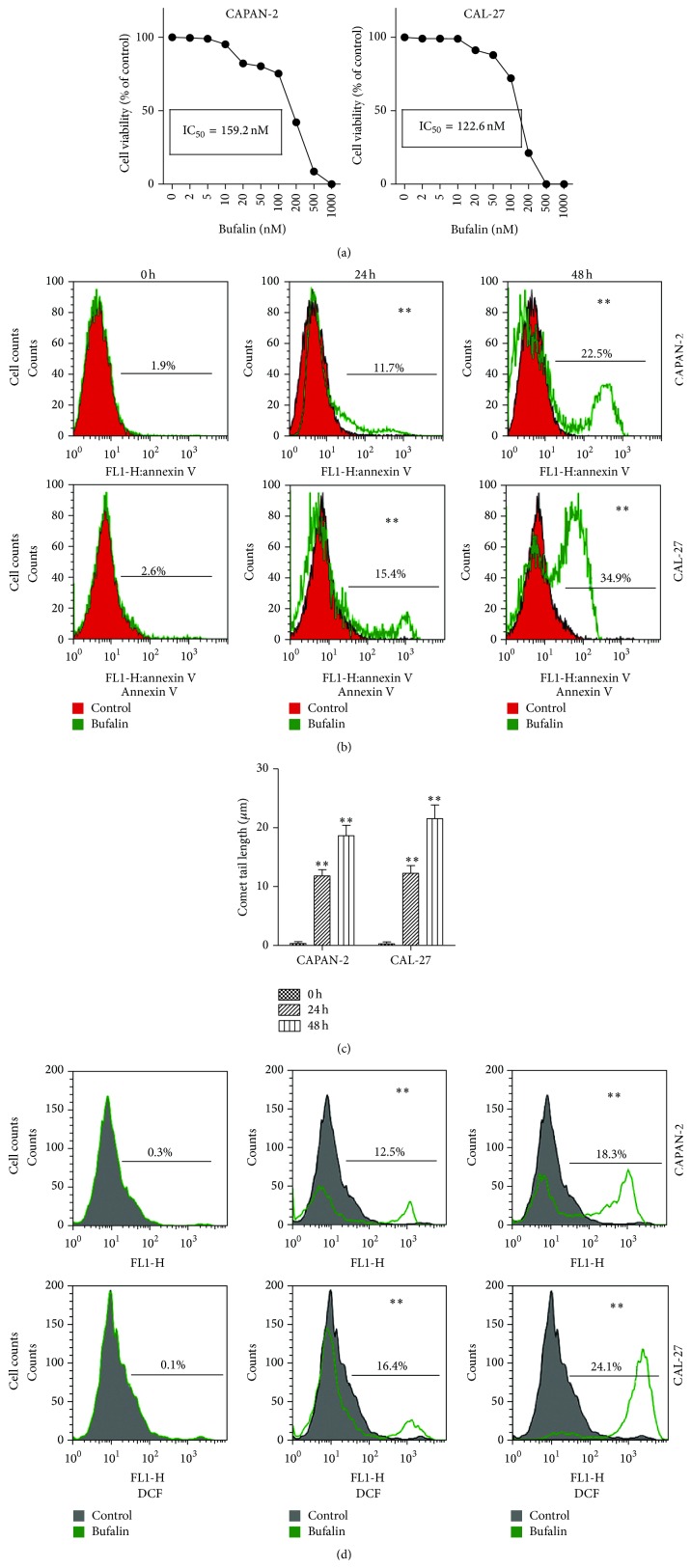
Bufalin increases intracellular ROS production and induces cell death and apoptosis in CAPAN-2 and CAL-27 cells. (a) CAPAN-2 and CAL-27 cells were treated with bufalin at the indicated concentrations for 24 h. Cell viability was determined by the MTT assay. The IC_50_ values were calculated using nonlinear regression. (b–d) CAPAN-2 and CAL-27 cells were treated with 100 nM bufalin for the indicated times. (b) Cell apoptosis determined by flow cytometry using the annexin V-FITC staining. (c) DNA damage determined by the comet assay. The comet tail length was indicative of the extent of DNA damage. (d) Intracellular ROS levels determined by flow cytometry using the DCFH-DA staining. ^*∗∗*^ *p* < 0.01 versus control (0 h).

We first tested the effects of bufalin on cell viability of CAPAN-2 and CAL-27 cells using the MTT assay. After treatment with bufalin for 24 h, the cell viability of CAPAN-2 and CAL-27 cells decreased with increasing bufalin concentrations (Figure 1(a)). The IC_50_ values of bufalin were calculated to be 159.2 nM and 122.6 nM for CAPAN-2 and CAL-27 cells, respectively. We then studied the effects of bufalin on cell apoptosis by flow cytometry using the annexin V-FITC staining. When CAPAN-2 and CAL-27 cells were treated with 100 nM bufalin for 24 and 48 h, the percentage of apoptotic cells significantly increased with treatment time (Figure 1(b)). Moreover, 100 nM bufalin significantly induced DNA damage in the two cell lines as revealed by the comet assay (Figure 1(c)), providing further evidence for bufalin-induced apoptosis. To investigate the possible mechanisms involved, we determined the intracellular ROS levels using the ROS detection reagent DCFH-DA. Significantly higher ROS levels were detected in bufalin-treated CAPAN-2 and CAL-27 cells (Figure 1(d)), suggesting that mitochondrial oxidative damage may contribute to bufalin-induced cell apoptosis.

### 3.2. Bufalin Downregulates hTERT Expression in CAPAN-2 and CAL-27 Cells

**Figure 2:**
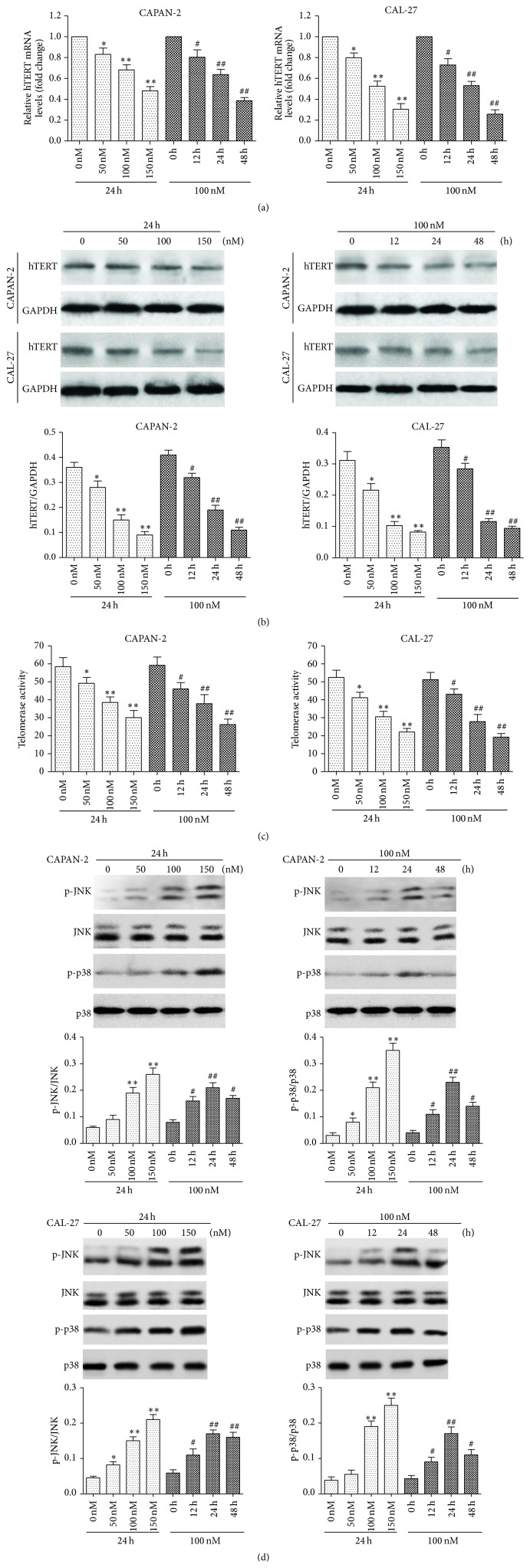
Bufalin downregulates hTERT expression in CAPAN-2 and CAL-27 cells. CAPAN-2 and CAL-27 cells were treated with bufalin at the indicated concentrations for the indicated times. (a) The hTERT mRNA expression determined by qRT-PCR. Data were normalized to *β*-actin and expressed as fold change relative to the untreated control. (b) The hTERT protein expression determined by western blot. Data were normalized to GAPDH. (c) The telomerase activity determined by the telomerase PCR enzyme-linked immunosorbent assay. (d) The protein levels of p-JNK, JNK, p-p38, and p38 determined by western blot. The activation of JNK and p38 was assessed by the p-JNK/JNK and p-p38/p38 ratios, respectively. ^*∗*^ *p* < 0.05, ^*∗∗*^ *p* < 0.01 versus control (0 nM). ^#^ *p* < 0.05, ^##^ *p* < 0.01 versus control group (0 h).

hTERT has been shown to inhibit intracellular ROS production, improve mitochondrial function, and prevent ROS-mediated apoptosis in cancer cells [21, 22]. To find out whether hTERT plays a role in bufalin-induced ROS production and cell apoptosis in CAPAN-2 and CAL-27 cells, we assessed the mRNA (Figure 2(a)) and protein expression (Figure 2(b)) of hTERT using qRT-PCR and western blot, respectively. We found that bufalin significantly decreased the hTERT mRNA and protein expression as well as the telomerase enzymatic activity in CAPAN-2 and CAL-27 cells in a dose and time-dependent manner (Figure 2(c)). To clarify the possible role of MAPK in telomerase regulation, we assessed the activation of JNK and p38-MAPK by measuring the protein levels of p-JNK and p-p38 using western blot. Our data showed that the p-JNK/JNK and p-p38/p38 ratios in bufalin-treated CAPAN-2 and CAL-27 cells were significantly higher than that in untreated cells (Figure 2(d)). These findings suggested that JNK and p38 might be involved in the hTERT downregulation by bufalin.

### 3.3. hTERT Silencing Triggers Mitochondria-Dependent Apoptosis of CAPAN-2 and CAL-27 Cells

**Figure 3:**
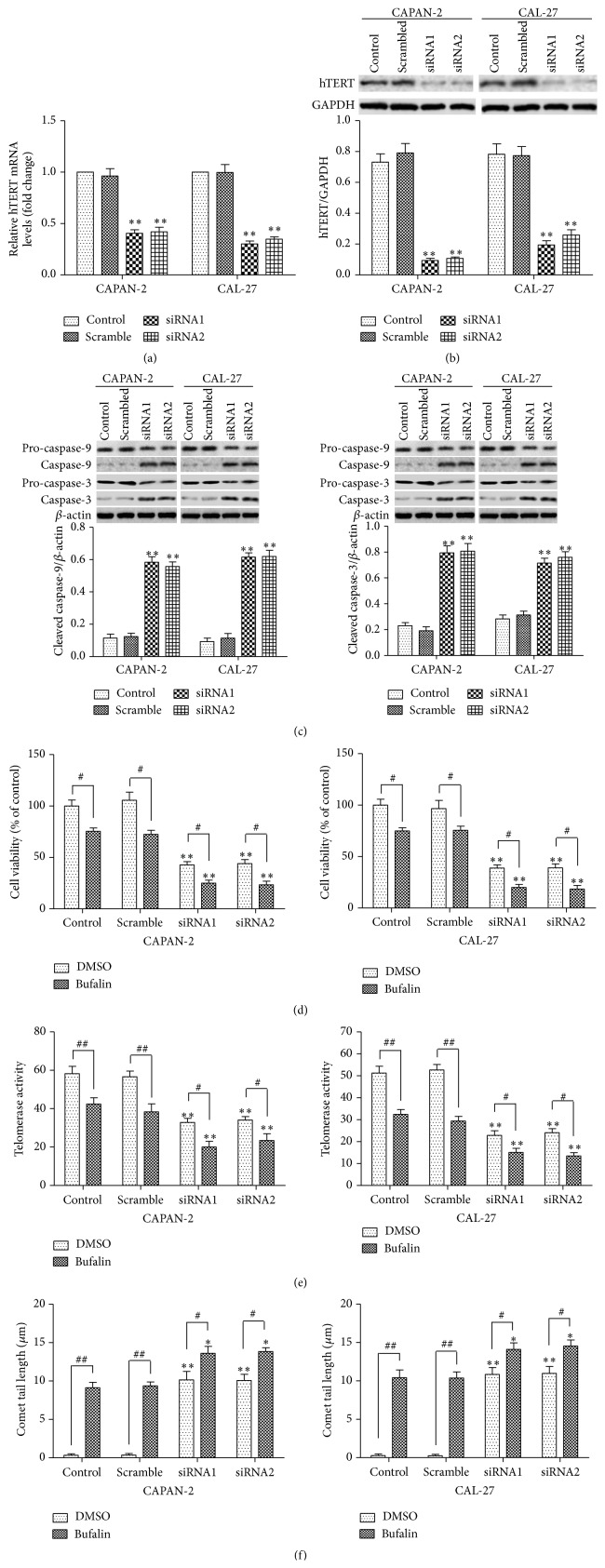
hTERT silencing triggers mitochondria-dependent apoptosis of CAPAN-2 and CAL-27 cells. (a–c) CAPAN-2 and CAL-27 cells were transfected with scrambled siRNA, hTERT siRNA1, or hTERT siRNA2 for 48 h. The nontransfected cells were included for comparison. (a) The hTERT mRNA expression determined by qRT-PCR. Data were normalized to *β*-actin and expressed as fold change relative to the control. (b) The hTERT protein expression determined by western blot. Data were normalized to GAPDH. (c) The protein levels of procaspase-9/-3 and cleaved caspase-9/-3 determined by western blot. Data were normalized to *β*-actin. ^*∗∗*^ *p* < 0.01 versus control. (d–f) CAPAN-2 and CAL-27 cells transfected with scrambled siRNA, hTERT siRNA1, or hTERT siRNA2 were treated with 100 nM bufalin or vehicle alone for 24 h. The nontransfected cells were included for comparison. (d) Cell viability determined by the MTT assay. (e) The telomerase activity determined by the telomerase PCR enzyme-linked immunosorbent assay. (f) DNA damage determined by the comet assay. The comet tail length was indicative of the extent of DNA damage. ^*∗∗*^ *p* < 0.01 versus control with the corresponding DMSO or bufalin treatment. ^#^ *p* < 0.05, ^##^ *p* < 0.01.

We subsequently investigated the role of hTERT in CAPAN-2 and CAL-27 cell apoptosis using hTERT silencing by siRNA. The hTERT mRNA (Figure 3(a)) and protein expression (Figure 3(b)) and the telomerase activity (Figure 3(e)) were effectively suppressed by transfection with hTERT siRNA1 or hTERT siRNA2. Compared with cells transfected with the scramble siRNA, cells transfected with hTERT siRNAs showed significantly increased levels of cleaved caspase-9/-3 (Figure 3(c)). Caspase-9 is an initiator caspase of the mitochondrial pathway of apoptosis. Once initiated caspase-9 goes on to cleave procaspase-3 and procaspase-7. Thus these data suggested that hTERT silencing in CAPAN-2 and CAL-27 cells activates the mitochondria-dependent pathway of apoptosis. Further studies showed that hTERT silencing by siRNA significantly decreased cell viability (Figure 3(d)) and increased DNA damage (Figure 3(f)), confirming that hTERT silencing induces mitochondria-dependent apoptosis. Treatment of untransfected or scrambled siRNA-transfected cells with 100 nM bufalin for 24 h resulted in significantly decreased telomerase activity and cell viability and increased DNA damage (Figures 3(d)–3(f)). Interestingly, bufalin treatment of hTERT siRNA1 or siRNA2-transfected cells led to further decreased telomerase activity and cell viability and further increased DNA damage (Figures 3(d)–3(f)). These effects of bufalin were presumably mediated by further downregulation of the hTERT expression in hTERT siRNA1 or siRNA2-transfected cells.

### 3.4. Blocking the JNK/p38 Pathway Reverses Bufalin-Induced hTERT Downregulation in CAPAN-2 and CAL-27 Cells

**Figure 4:**
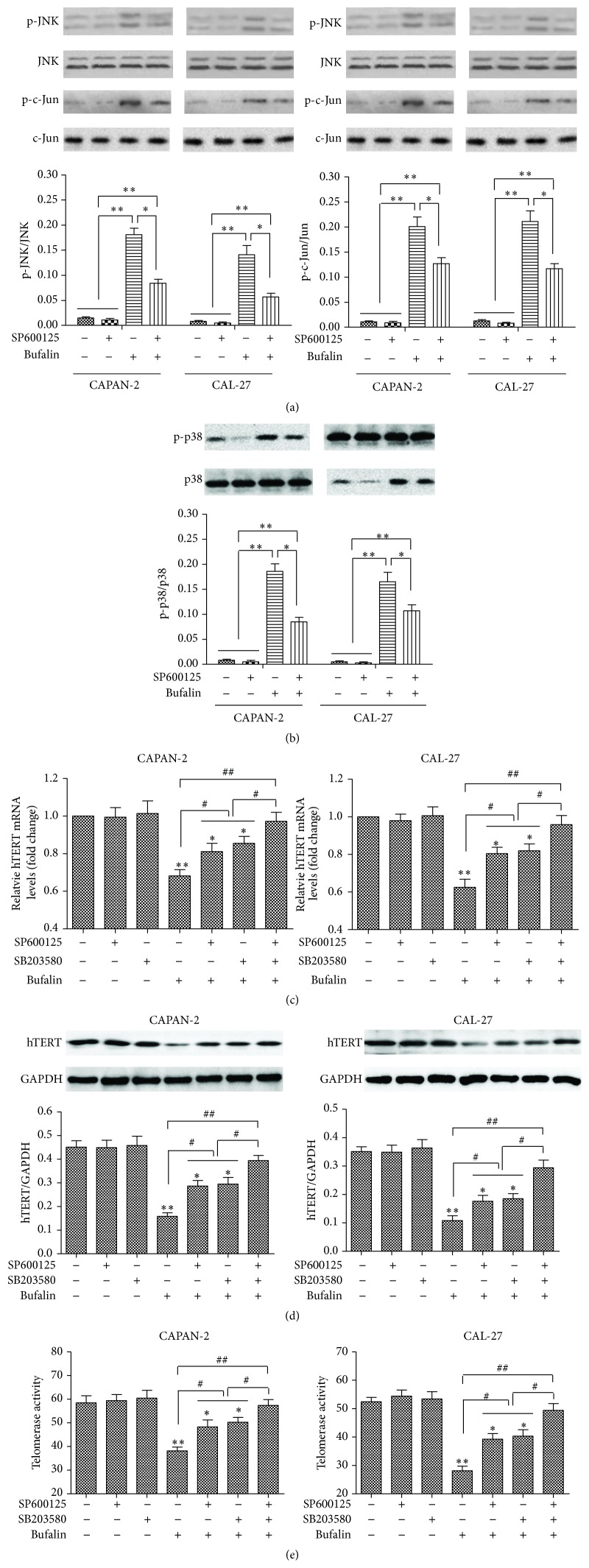
Blocking the JNK/p38 pathway reverses bufalin-induced hTERT downregulation in CAPAN-2 and CAL-27 cells. CAPAN-2 and CAL-27 cells were treated with 5 *μ*M SP600125 or 10 *μ*M SB203580 for 1 h and subsequently incubated with 100 nM bufalin for 24 h. Vehicle alone served as control. (a-b) The protein levels of p-JNK, JNK, p-c-Jun, and c-Jun (a) and p-p38 and p38 (b) were determined by western blot. Results of densitometric analysis are normalized to the correlative unphosphorylated protein. ^*∗*^ *p* < 0.05, ^*∗∗*^ *p* < 0.01. (c) The hTERT mRNA expression determined by RT-PCR. Data were normalized to *β*-actin and expressed as fold change relative to the control. ^*∗*^ *p* < 0.05, ^*∗∗*^ *p* < 0.01 versus the control, ^#^ *p* < 0.05, ^##^ *p* < 0.01. (d) The hTERT protein expression determined by western blot. Data were normalized to GAPDH. ^*∗*^ *p* < 0.05, ^*∗∗*^ *p* < 0.01 versus the control, ^#^ *p* < 0.05, ^##^ *p* < 0.01. (e) The telomerase activity determined by the telomerase PCR enzyme-linked immunosorbent assay. ^*∗*^ *p* < 0.05, ^*∗∗*^ *p* < 0.01 versus the control, ^#^ *p* < 0.05, ^##^ *p* < 0.01.

Since our data have shown that bufalin increases the phosphorylation of JNK and p38-MAPK in CAPAN-2 and CAL-27 cells, we speculated that the JNK/p38 pathway is involved in the hTERT regulation by bufalin. To test this hypothesis, we studied the effects of the JNK inhibitor SP600125 and the p38-MAPK inhibitor SB203580 on hTERT expression in bufalin-treated cells. CAPAN-2 and CAL-27 cells were treated with 5 *μ*M SP600125 (Santa Cruz, USA) or 10 *μ*M SB203580 (Santa Cruz) for 1 h and subsequently incubated with 100 nM bufalin for 24 h. The inhibition of JNK and p38-MAPK activities was confirmed by decreased phosphorylation of c-Jun (Figure 4(a)) and p38 (Figure 4(b)), respectively. Bufalin treatment alone significantly downregulated hTERT mRNA and protein expression, leading to decreased telomerase activity (Figures 4(c)–4(e)). This bufalin-induced hTERT downregulation was partially prevented by treatment with SP600125 or SB203580 alone and completely reserved by combined treatment with the two inhibitors (Figures 4(c)–4(e)). Therefore, the activation of the JNK/p38 pathway is required for the hTERT regulation by bufalin in CAPAN-2 and CAL-27 cells.

## 4. Discussion

In the present study, we demonstrated that bufalin reduced the viability of CAPAN-2 human pancreas cancer cells and CAL-27 human oral cancer cells with potent IC_50_ values of 159.2 nM and 122.6 nM, respectively. Moreover, bufalin increased ROS production, caused DNA damage, and induced apoptosis in these two cell lines. These effects of bufalin were mediated by the downregulation of hTERT expression, which activated the mitochondria-dependent pathway of apoptosis involving caspase-9/-3 activation. Furthermore, the hTERT regulation by bufalin in CAPAN-2 and CAL-27 cells was mediated by the activation of the JNK/p38 pathway. Therefore, bufalin may have therapeutic potential for the treatment of pancreas and oral cancers.

Bufalin has been reported to induce apoptosis through activation of the mitochondria-dependent pathway in a number of cancer cells [13–15]. Specifically, a recent study showed that bufalin inhibited CAL-27 cell growth with an IC_50_ value of 125 nM after 24 h treatment [27], which was very close to the IC_50_ value of 122.6 nM observed in this study. The same study also reported that bufalin induced cell cycle arrest at the G0/G1 phase, decreased Bcl-2 expression, increased cytochrome c, Apaf-1, and AIF expression, and induced caspase-9/-3-dependent apoptosis in CAL-27 cells, indicating that bufalin kills CAL-27 cells through activation of the mitochondrial pathway of apoptosis. However, the detailed molecular mechanisms involved are not fully understood. In the present study, we observed increased ROS production and DNA damage in bufalin-treated CAL-27 cells, providing further evidence that mitochondrial dysfunction is involved in bufalin-induced CAL-27 cell apoptosis.

Most importantly, in the present study, we identified hTERT as the key mediator of bufalin-induced apoptosis by the mitochondrial pathway. Mitochondrial hTERT is known to decrease mitochondrial ROS production and protect against mitochondrial damage by binding to mitochondrial DNA [23]. In the present study, hTERT silencing in CAPAN-2 and CAL-27 cells resulted in the activation of caspase-9/-3 and DNA fragmentation, which was consistent with induction of the mitochondrial pathway of apoptosis. It requires further studies to find out whether bufalin exerts antitumor effects in other cancer cell types via regulation of hTERT expression.

Previous studies have shown that bufalin exerts antitumor effects through inhibition of AKT [5, 27], heat shock protein 27 (Hsp27) [28, 29], GSK3beta/beta-catenin/E-cadherin [5], NF-kB [12], and matrix metalloproteinase-2/-9 [12] signaling pathways in various cancer cells. In the present study, we found that bufalin downregulates hTERT expression via the activation of the JNK/p38 pathway, providing new insights into the mechanisms underlying bufalin's anticancer activity.

Recently, novel bufalin formulations have shown promising efficacy in xenograft tumor models [7, 30, 31]. Randomized, double-blind, placebo-controlled human trials are now required to assess the therapeutic value of bufalin in various cancers.

## Supplementary Material

Supplemental Figure 1: CAPAN-2 and CAL-27 cells were treated with 100 nM bufalin for the indicated times. DNA damage determined by the comet assay.
Supplemental Figure 2: CAPAN-2 and CAL-27 cells transfected with scrambled siRNA, hTERT siRNA1, or hTERT siRNA2 were treated with 100 nM bufalin or vehicle alone for 24 h. The non-transfected cells were included for comparison. DNA damage determined by the comet assay.
