# Supplementary material for: Bufalin Induces Mitochondria-Dependent Apoptosis in Pancreatic and Oral Cancer Cells by Downregulating hTERT Expression via Activation of the JNK/p38 Pathway
Source: Evid Based Complement Alternat Med. 2015 Dec 10;2015:546210. doi: 10.1155/2015/546210 (PMC4689913; doi:10.1155/2015/546210)

Supplemental Figure 1

CAPAN-2 and CAL-27 cells were treated with 100 nM bufalin for the indicated times. DNA damage determined by the comet assay.

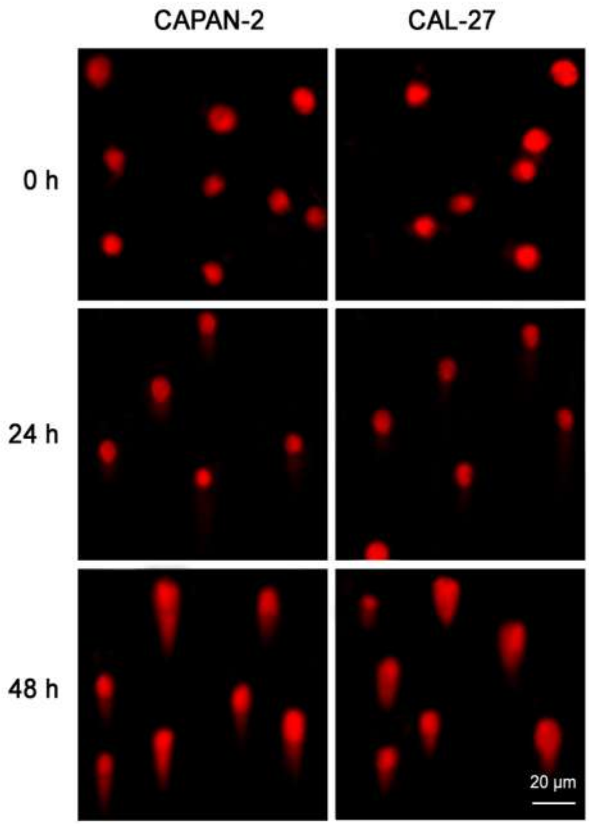

Supplemental Figure 2

CAPAN-2 and CAL-27 cells transfected with scrambled siRNA, hTERT siRNA1, or hTERT siRNA2 were treated with 100 nM bufalin or vehicle alone for 24 h. The non-transfected cells were included for comparison. DNA damage determined by the comet assay.

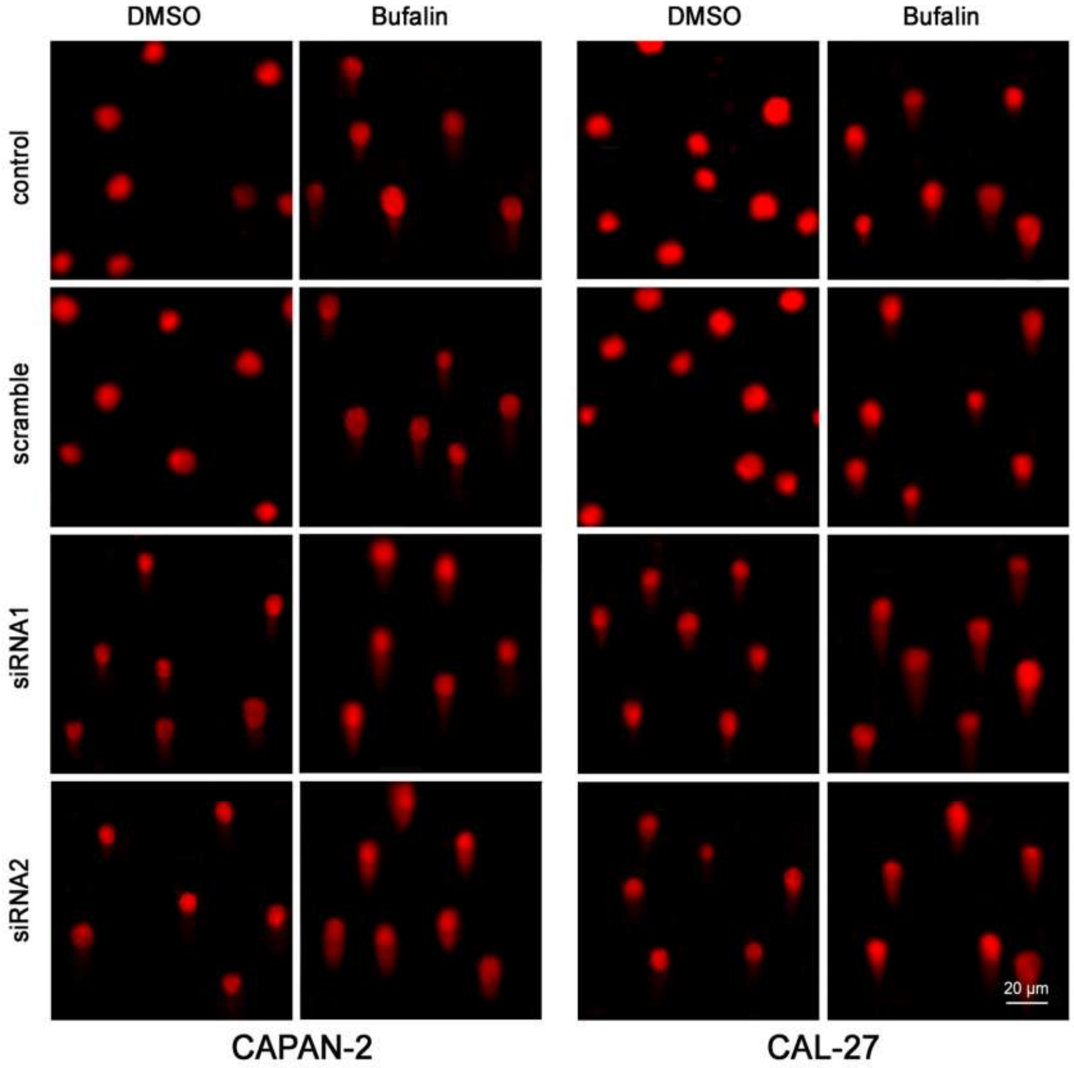

Supplement: Supplementary file 1 — Supplemental Figure 1: CAPAN-2 and CAL-27 cells were treated with 100 nM bufalin for the indicated times. DNA damage determined by the comet assay. Supplemental Figure 2: CAPAN-2 and CAL-27 cells transfected with scrambled siRNA, hTERT siRNA1, or hTERT siRNA2 were treated with 100 nM bufalin or vehicle alone for 24 h. The non-transfected cells were included for comparison. DNA damage determined by the comet assay. [file 546210.f1.pdf]
